# Abaloparatide Real‐World Patient Experience Study

**DOI:** 10.1002/jbm4.10457

**Published:** 2021-02-04

**Authors:** Deborah T Gold, Richard Weiss, Tammy Beckett, Chad Deal, Robert S Epstein, Andrew L James, Jacqueline M Kernaghan, Mahshid Mohseni, Michael Spiegel, Tamara Vokes, Jenna Roberts, Tom Bailey, Yamei Wang, Setareh A Williams

**Affiliations:** ^1^ Departments of Psychiatry & Behavioral Sciences and Sociology Duke University Medical Center Durham NC USA; ^2^ Global Medical Affairs Radius Health, Inc. Waltham MA USA; ^3^ Department of Orthopaedics Orthopaedic Associates of Grand Rapids Research and Education Institute Grand Rapids MI USA; ^4^ Center for Osteoporosis and Metabolic Bone Disease, Department of Rheumatology The Cleveland Clinic Foundation Cleveland OH USA; ^5^ Epstein Health, LLC Woodcliff Lake NJ USA; ^6^ Adult Health CNS Proactive Orthopaedics Proactive Orthopaedics at Columbia Orthopaedic Groups LLP Columbia MO USA; ^7^ Osteoporosis Center of Delaware County Prospect Health Access Network Springfield PA USA; ^8^ Department of Medicine, Division of Bone and Mineral Diseases Washington University School of Medicine St Louis MO USA; ^9^ WCMG Rheumatology Western Connecticut Health Network Danbury CT USA; ^10^ Department of Medicine, Section of Endocrinology University of Chicago Chicago IL USA; ^11^ Observational Research Adelphi Real World Bollington, Macclesfield UK; ^12^ Biometrics Radius Health, Inc. Waltham, MA USA; ^13^ Health Economics and Outcomes Research Radius Health, Inc. Waltham, MA USA

**Keywords:** ANABOLICS, HEALTH SERVICES RESEARCH, OSTEOPOROSIS

## Abstract

Despite the availability of various osteoporosis treatments, adherence remains suboptimal. One contributing factor may be patient experience with therapy. This US, multicenter, combined retrospective chart review and patient questionnaire study included postmenopausal women at high risk for fracture and is the first study to describe real‐world patient experience with abaloparatide (ABL) injection. Eight geographically diverse secondary care sites in the United States participated (*n* = 193). Mean ± SD age was 67.4 ±8.62 years. Most patients (86%) were satisfied with the ABL regimen, especially with ease of preparation (82%), ease of storage (87%), and storage convenience (89%), an attribute 83% of the patients thought was important. The majority of patients reported complete satisfaction with the ABL regimen allowing for their ability to conduct daily activities (85%) and convenience to fit into their daily schedule (84%). All reported taking ABL as directed, by injection in the lower abdomen, and 83% of patients reported medium or high adherence. Patients were satisfied with the needle size (76% completely satisfied), and 93% reported never deliberately missing a dose. Although injecting medication (18%) and higher out‐of‐pocket costs (17%) were deemed the most bothersome attributes, the majority (69%) noted their healthcare team understands how osteoporosis impacts their lives. In multivariable analyses, ease of preparation (OR = 2.62; 95% CI, 1.01–6.81; *p* = 0.048) and fracture history (OR = 1.72; 95% CI, 1.03–2.86; *p* = 0.037) were significantly associated with overall satisfaction. Ease of preparation was a predictor of higher satisfaction with treatment convenience (coefficient = 13.60; 95% CI, 8.08–19.12; *p* = 0.00). Remembering to take the medication was a significant predictor of self‐reported adherence (OR = 16.66; 95% CI, 3.30–84.24; *p* = 0.001). In conclusion, the majority of patients were satisfied with ABL and found it convenient/easy to prepare and store. High self‐reported adherence may be associated with positive patient experience including ease of use and adequate support from healthcare providers. © 2020 The Authors. *JBMR Plus* published by Wiley Periodicals LLC. on behalf of American Society for Bone and Mineral Research.

## Introduction

Osteoporosis is a systemic disease leading to a progressive decrease in BMD, decreased bone strength, and increased risk of skeletal fractures. According to the National Osteoporosis Foundation, approximately 10 million Americans have osteoporosis and 44 million more have low bone mass (osteopenia).^(^ ^1^ ^)^ An estimated two million osteoporotic fractures each year in the United States are associated with $19 billion in healthcare costs. These figures are predicted to grow by 2025 to approximately three million fractures and $25 billion annually as the population of older Americans increases.^(^ ^2^ ^)^

Historically, osteoporosis patients in receipt of pharmacological intervention have been treated with antiresorptive agents (bisphosphonates [oral, i.v.], denosumab, estrogens, calcitonin) that reduce osteoclast bone resorption and thus prevent more bones from being broken down.^(^ ^3^ ^)^ Anabolic drugs, which can add bone and potentially improve bone microarchitecture, have become available as an additional treatment option. These include teriparatide (TPTD) (Forteo; Eli Lilly and Co., Indianapolis, IN, USA), a first‐in‐class anabolic agent receiving US Food and Drug Administration (FDA) approval in 2002, abaloparatide (ABL) (Tymlos; Radius Health, Inc., Waltham, MA, USA), approved by the FDA in 2017, and romosozumab (Evenity; Amgen, Inc., Thousand Oaks, CA, USA) approved in 2019. These drugs are indicated for the treatment of postmenopausal osteoporosis in women at high risk for fracture, defined as a history of osteoporotic fracture, multiple risk factors for fracture, or patients who have failed or are intolerant to other available osteoporosis therapy. Although ABL and TPTD are self‐administered by daily s.c. injection, the more recently approved romosozumab requires monthly injections at a healthcare professional's office.

The approval of ABL included consideration of results at 18 months from the landmark ACTIVE trial^(^ ^4^ ^)^ and the first 6 months of the ACTIVExtend trial, which demonstrated consistent significant and rapid reductions in the risk of vertebral and nonvertebral fractures regardless of age, years since menopause, presence or absence of prior fracture, and BMD.^(^ ^5^ ^)^

In addition to clinical outcomes reported from randomized controlled trials (RCTs), the real‐world patient experience is important to understand and inform treatment decisions.^(^ ^6^, ^7^, ^8^ ^)^ Patients treated in usual care settings may not adhere or respond to a given therapy as well as is noted within the controlled and highly selected environment of protocol‐driven RCTs. Because osteoporosis and the resulting complications of fractures impact numerous dimensions of patients' quality of life (including physical functioning, emotional functioning, pain, and activity levels), it may be anticipated that real‐world studies may amplify the need to adhere to therapy.^(^ ^8^ ^)^ However, poor adherence to treatment has also been cited as a consistent and significant problem in the real‐world care of osteoporosis, resulting in reduced therapeutic benefit.^(^ ^9^ ^)^

A range of causal factors for poor adherence to therapy has been identified, including patient‐related factors (eg, disease understanding) and medication‐related factors (eg, administration frequency, dosing requirements, etc.).^(^ ^10^, ^11^ ^)^ Patients who express lower levels of treatment satisfaction are less likely to persist with therapy.^(^ ^12^ ^)^ In view of this, educational interventions to ensure adequate understanding of osteoporosis and its risks, improved patient and physician communication on disease and its management, ease and convenience of medication administration, and consideration of patient preferences are all recognized as important contributing factors to successful treatment initiation, adherence, and health outcomes.^(^ ^13^, ^14^, ^15^ ^)^ Real‐world data on patient experience with ABL may contribute to the existing literature and help in the identification of appropriate patients for treatment.

The primary objective of this study was to describe the experience of patients who have initiated treatment with ABL (anabolic treatment‐naïve patients, cohort I) or have switched from another anabolic agent, ie, TPTD (anabolic treatment‐experienced patients, cohort II). The experience assessment included treatment satisfaction, ease of use, and adherence. Secondary objectives included characterization of patients initiating ABL in a real‐world practice setting and documentation of variation of attribute preference by patient characteristics.

## Patients and Methods

### Study design

This was a US, multicenter, observational study including a cross‐sectional patient survey and a linked electronic Case Report Form (eCRF) comprising patient level data abstracted from the medical records. All treatment decisions for participating patients were made independent of the research study. The study protocol and supporting documents, including the consent form, patient questionnaire, and the eCRF were submitted to an Institutional Review Board (IRB) for written approval in accordance with the site‐specific policies. In the absence of a local policy, a central IRB (The Western Institutional Review Board) was used. IRB approval was received at every site prior to the study execution. The study was conducted in accordance with the Guidelines for Good Pharmacoepidemiology Practices issued by the International Society for Pharmacoepidemiology.

### Study population

The study included osteoporosis patients who were receiving treatment with ABL prior to study enrollment from geographically diverse community and academic‐affiliated physician practices.

### Inclusion/exclusion criteria

**Fig 1:**
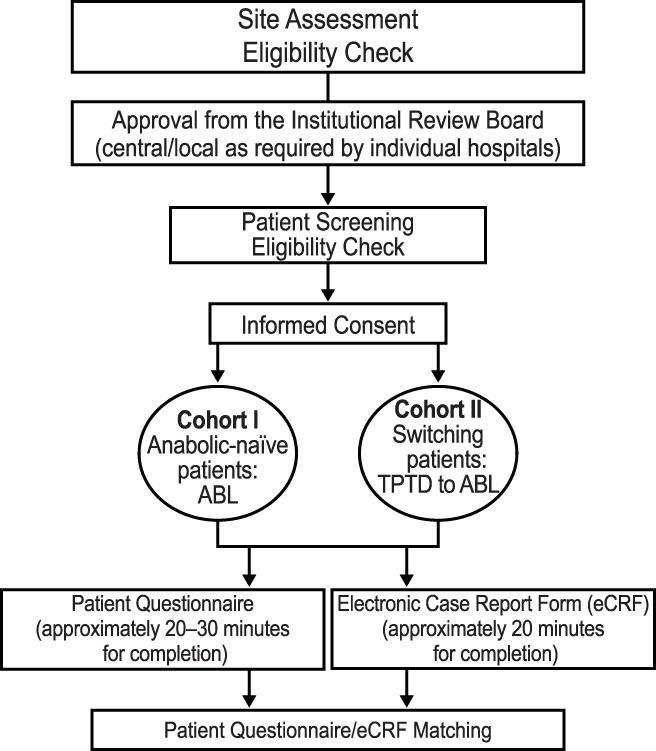
Overall study design. ABL = abaloparatide; eCRF = electronic Case Report Form; TPTD = teriparatide.

Specific criteria for inclusion were postmenopausal women with a physician‐confirmed diagnosis of osteoporosis at high risk for fracture per their physicians' assessment. Patients could have been on ABL as their first anabolic treatment (anabolic treatment‐naïve, cohort I) or have had their medication switched from TPTD to ABL (anabolic treatment‐experienced, cohort II) (Fig. 1). Patients were required to have been on treatment for approximately a month prior to study enrollment with no upper limit of duration of use and may have had prior exposure to other osteoporosis drug classes. Additional inclusion criteria were completion of informed consent, ability to read and understand English, and outpatient status at the time of enrollment. Patients participating in any clinical trials and those with Paget's disease, preexisting hypercalcemia, primary hyperparathyroidism, urolithiasis, and hypercalciuria were excluded.

### Study measures

Data were captured from two sources: a self‐completed paper‐based patient questionnaire (Supplemental Item 1) and a site‐completed standardized eCRF (Supplemental Item 2).

### Patient questionnaire

Patients were requested to independently complete a questionnaire containing four self‐reported instruments to assess treatment satisfaction, adherence, and functioning/health‐related quality of life (HRQoL). Additional questions were included to assess areas of interest for which there are no validated measures.

Treatment satisfaction was measured with the psychometrically validated treatment satisfaction questionnaire for medication (TSQM‐9), a nine‐item questionnaire containing effectiveness, convenience, and global satisfaction domains.^(^ ^16^, ^17^ ^)^ The TSQM‐9 domain scores range from 0 to 100, with higher scores representing higher satisfaction. There is no threshold for what is considered a “high” satisfaction score.

Treatment adherence was assessed with the eight‐item Osteoporosis‐Specific Morisky Medication Adherence Scale (OS‐MMAS).^(^ ^18^ ^)^ The scale has acceptable psychometric properties for assessing medication adherence in postmenopausal women prescribed therapy for osteoporosis. Each of the eight items of the disease‐specific OS‐MMAS captures a specific medication‐taking behavior. Response categories are yes/no for items 1 to 7 and a five‐point Likert response for the last item. The OS‐MMAS scores can range from 0 to 8 and have been categorized into three levels: high adherence (score = 8), medium adherence (6 to <8), and low adherence (<6).

HRQoL was assessed using the Osteoporosis Assessment Questionnaire Short Version (OPAQ‐SV) with three domains: physical functioning, emotional status, and back pain, with higher values indicating better function.^(^ ^19^ ^)^ The short version of the scale was used to minimize the respondent burden. In addition to the above, the five‐level version of the EuroQol‐5D (EQ‐5D‐5L), including the Visual Analogue Scale (VAS), was used to measure patients' self‐report of overall health status.^(^ ^20^ ^)^ Last, other factors including patient perception of disease severity, understanding/awareness of osteoporosis treatment, reasons for poor adherence, and views on disease management were explored.

### Case report form

For eligible patients who completed the questionnaire, the physician or a delegated healthcare professional from the sites provided patient data from the medical records including, but not limited to, osteoporosis disease confirmation, diagnostic/treatment history, information on comorbid conditions and concomitant medication, outcomes data including fracture history, changes in BMD, and bone turnover markers (BTMs). Patient risk status was determined using medical and treatment history including items from the Fracture Risk Assessment Tool (FRAX®)^(^ ^21^ ^)^ (eg, smoking status, alcohol intake, family history, BMI, etc.) to the extent available. Data abstracted by site staff from patient medical records were entered into an eCRF hosted on secure, password‐protected, electronic data capture (EDC) software. The software was certified as being compliant with the FDA's 21 CRF Part 11 regulation. Data storage was Health Insurance Portability and Accountability Act (HIPAA)‐compliant.

### Statistical analyses

The primary objective of this study was to provide descriptive statistics without a priori hypotheses; therefore, formal sample size calculations were not used. The study size was chosen in consideration of both the practical constraints and precision of descriptive statistics. In the current study sample, no more than 10 independent variables were used, following a rule of thumb that 20 observations are required for each covariate.^(^ ^22^ ^)^

All analyses were conducted using Stata 15.1 (StataCorp LLC, College Station, TX, USA). To address the primary objective and most of the secondary objectives, descriptive statistics were produced for variables of interest. Regression models were used to evaluate predictors for treatment satisfaction, adherence, and ease of use with ABL. All regression models were produced twice for each measure of patient risk assessed by their modified FRAX score and separately by prior fracture history. Furthermore, subgroup analyses were carried out for the population of patients who found injection as the most bothersome treatment attribute. Independent variables used as predictors were carefully selected from the data, guided by disease knowledge and the published literature.^(^ ^10^, ^11^, ^12^, ^13^, ^14^, ^15^, ^16^ ^)^ The regression models used (ordinary least squares [OLS], generalized linear model [GLM], etc.) were appropriate to the form of the outcome variable collected. For each independent variable in the regression, the estimated coefficient, standard error, test statistic, *p* value, and 95% confidence interval (CI) are reported.

### Missing data

Some degree of missing data was expected due to incompleteness of patient medical records and patients potentially missing or deliberately skipping certain questions in the questionnaire. Missing data were imputed if appropriate per each instrument, otherwise they remained missing. A completion threshold of 50% was set and patient questionnaires missing 50% or more entries were considered to have withdrawn from the study.

## Results

### Data source

A total of 193 patients were recruited from eight geographically diverse secondary sites in the United States (Table 1). Thirty‐eight percent of the sites were academic and/or teaching hospitals, whereas others were community‐based practice settings. Approximately a quarter of the physicians were family practitioners or primary care physicians, and the remaining were distributed evenly across various specialties of endocrinology (17%), gynecology (17%), rheumatology (14%), and orthopedic surgery (12%). Three‐quarters of the patients were new to anabolic treatment (cohort I) and one‐quarter were anabolic switches (cohort II).

**Table 1:** Final Sample, Split by Study Sites

Site	Cohort I	Cohort II	Total
Columbia Orthopaedic Group	24	21	45
Swedish Center for Comprehensive Care	30	3	33
Crozer‐Keystone Health System	20	12	32
Orthopaedic Associates of Michigan	21	2	23
Cleveland Clinic	18	2	20
Washington University	16	4	20
University of Chicago	13	0	13
Western Connecticut Health Network	5	2	7
Total	147	46	193

Values are the number of patients. cohort I = anabolic naïve; cohort II = anabolic switches.

### Patient characteristics

The mean ± SD age was 67.4 ± 8.62 years with a median BMI of 23.7 (interquartile range [IQR] 20.8, 26.9). The majority of patients on ABL were considered to have severe (54.4%) or very severe (25.9%) osteoporosis based on their physician's assessment.

### Medical and treatment history

The median time since diagnosis of osteoporosis was 2.67 years (IQR 1.15, 8.07). Forty‐four percent of the patients were diagnosed as a result of routine screening and one‐quarter were diagnosed at the time of a fragility fracture. Use of a central dual‐energy X‐ray absorptiometry (DXA) scan was most often indicated as a risk assessment tool (79%) used by the treating physician. Other tools used by the participating physicians included conventional X‐ray (10%) and the FRAX risk assessment tool (6%). In the year prior to ABL initiation, 34% of patients had at least one vertebral and/or nonvertebral fracture. Other indicators of fracture risk included having a history of falls in the month preceding study enrollment (7%), parental history of hip fracture (23%), and smoking (30%; 6% current and 24% former smokers) (Table 2). Although alcohol use is associated with higher risk of fractures, the study population self‐reported a low level of intake (77% drank four times per month or less and 93% had two or fewer units on a typical day when they drank) (Table 2).

**Table 2:** Patient Characteristics

Patient demographics	Patients surveyed (*n* = 193)
Age (years), mean ± SD	67.4 ± 8.62
Age range (years), minimum, maximum	33, 92
Level of education, *n* (%)	
Less than high school	3 (2)
High school diploma or GED	65 (34)
College degree (2‐year – Associate's)	26 (13)
College degree (4‐year – Bachelor's)	44 (23)
Graduate degree or higher	43 (22)
Trade school/certificate program	7 (4)
Other	2 (1)
Missing data	3 (2)
Current employment status, *n* (%)	
Working full‐time	39 (20)
Working part‐time	22 (11)
On long‐term sick leave	1 ( 1)
Retired	107 (55)
Homemaker	13 (7)
Unemployed	8 (4)
Do not know	1 (1)
Disability allowance	1 (1)
Not stated	2 (1)
Frequency of physical activity, *n* (%)	
Not active	11 (6)
Once per week	17 (9)
2–3 times per week	50 (26)
≥4 times per week	113 (59)
Missing data	2 (1)
Fallen in the past month, *n* (%)	
Yes	14 (7)
No	178 (92)
Missing data	1 (1)
Smoking status, *n* (%)	
Current smoker	12 (6)
Previous smoker	46 (24)
Nonsmoker	134 (69)
Missing data	1 (1)
Frequency of alcohol consumption, *n* (%)	
0–1 time per month	108 (56)
2–4 times per month	40 (21)
2–3 times per week	25 (13)
≥4 times per week	17 (9)
Missing data	3 (2)
Family history of hip fractures, *n* (%)^a^	
Yes	44 (23)
No	138 (72)
Do not know	10 (5)
Not surveyed	1 (1)

^a^ Defined as either parent having ever had a hip fracture.

GED = General Educational Development.

The majority of patients (84.5%) did not report unnecessary delay between osteoporosis diagnosis and receipt of first osteoporosis medication. Of those who reported a delay, the following reasons for delay were noted: requirement to visit a specialist (41.4%); insurance coverage (34.5%); test results (27.6%); and physician decision to monitor disease progress (24.1%). Only 14.5% of the patients reported ever having a delay between prescription and receipt of medication that was longer than 1 day. The median reported delay was 3 days (IQR 3.0, 4.0). Of those who reported a delay, the following reasons for delay were noted: 3.6% reported incomplete prescription and 7.1% reported medication being out of stock; 89.3% were unsure of the reason for the delay. Overall, patients were on ABL for a median of 6 months prior to study enrollment with a range of 1 to 19 months. Sixty‐three percent (97/147 in cohort I and 24/46 in cohort II) of the study population were taking an osteoporosis treatment prior to initiation of ABL treatment. Weekly alendronate was the most common prior treatment (40%; 42/97 cohort I, 7/26 cohort II), and history of alendronate use was higher for patients new to anabolic therapy (43%) versus anabolic switches (27%). Other treatments used prior to ABL treatment initiation were zoledronic acid once yearly (18%; 19/97 cohort I, 3/26 cohort II), denosumab (12%; 7/97 cohort I, 8/26 cohort II), ibandronate monthly (9%; 10/97 cohort I, 1/26 cohort II), and alendronate daily (7%; 6/97 cohort I, 2/26 cohort II). Median duration of treatment preceding switching to ABL from TPTD was 19 months. The median time between discontinuation of therapy and initiation of anabolic treatment was 12 months for ABL. For patients who switched from TPTD to ABL, main reasons for change in treatment included mandatory formulary switch (30%), poor tolerability (20%), lack of treatment efficacy (9%), and hypercalcemia (7%).

### Patient management

For all patients, the scheduled mean duration for ABL use was 19.5 months (range 1.5–36.0 months). At the time of study initiation, 52% of patients were on ABL for <6 months, 27% for 6 to 12 months, and 21% for >1 year. The mean time since most recent DXA was 10 months. BTMs were primarily measured at the time of treatment initiation (35%) and for treatment monitoring (24%). Serum type 1 procollagen C‐terminal (P1CP) or N‐terminal (P1NP) (59%), serum total alkaline phosphate (58%), serum bone‐specific alkaline phosphate (26%), and urinary or serum collagen type 1 cross‐linked C‐telopeptide CTX (22%) were the most commonly used tests.

### Preliminary outcomes

#### Fracture incidence

From the time of ABL initiation, only two fractures (1%) were recorded (*n* = 193) over the duration of available follow‐up in this evaluation.

#### Change in BMD from baseline

Of the 142 patients with central DXA scans at diagnosis, mean ± SD *T* scores were −2.39 ± 0.79 (IQR = −2.9, −1.8), −2.09 ± 0.87 (IQR = −2.5, −1.6), −2.52 ± 0.96 (IQR = −3.2, −1.9), −2.11 ± 1.05 (IQR = −2.8, −1.4), and −3.53 ± 1.77 (IQR = −4.7, −1.5) at the femoral neck, total hip, lumbar spine, radius, and other areas (not including the mentioned categories), respectively.

## Patient reported experience with ABL

### Treatment satisfaction

**Fig 2:**
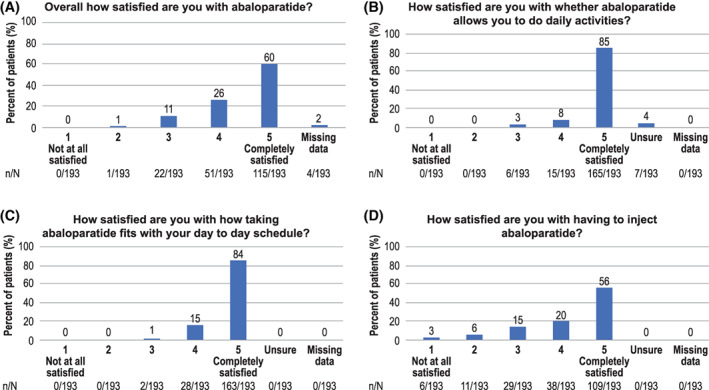
Patient satisfaction with abaloparatide. (A) Overall satisfaction. (B) Satisfaction with ability to do daily activities. (C) Satisfaction with compatibility with daily schedule. (D) Satisfaction with need to inject.

In response to the TSQM‐9, patients reported high levels of overall satisfaction with ABL (mean ± SD) (75 ± 19), satisfaction with treatment convenience (84 ± 15) and treatment effectiveness (74 ± 17). In addition to TSQM‐9, response to individual questions supported high satisfaction with ease of use (patient questionnaire‐QA1, 6–9) with the majority of patients reporting complete satisfaction with ease of storage (87%), preparation (82%), and travel (70%); 89% of patients indicated ABL is very easy/convenient to store, an attribute that 83% of the patients thought was important (patient questionnaire‐QA10‐QA11). Overall, 86% of patients were satisfied or completely satisfied with ABL (score of 4.5 on a scale of 1–5) (patient questionnaire‐QA5; Fig. 2A). The majority of patients reported complete satisfaction with the ABL regimen allowing for their ability to conduct daily activities (85%) (Fig. 2B) and the regimen's convenience to fit into their daily schedule (84%) (patient questionnaire‐QA1, 1 and 4; Fig. 2C), and 56% were completely satisfied with having to inject ABL (patient questionnaire‐QA1, 11) (Fig. 2D). Notably, patients also reported higher satisfaction with ABL compared with treatments prior to ABL (86% versus 51%). Satisfaction was driven by ease of use, convenience, as well as perception of treatment effectiveness. When asked about the best and worst features of treatment (patient questionnaire‐QA2a,b), patients noted the medication's ability to build bone (22%), reduce fracture risk (9%), and allow for normal activities of daily life (10%) as the most favorable features, whereas having to inject the medication (18%), injecting in the abdomen (7%), and higher out‐of‐pocket costs (17%) were deemed the most bothersome attributes.

### Adherence

According to the OS‐MMAS, 83% of the patients reported medium or high adherence with their treatment (patient questionnaire‐QB1‐8). On a scale of 0 to 8, approximately 40% reported high (score = 8), 44% medium (6 to <8), and 17% low adherence (<6). All reported taking ABL as directed, by injection in the lower abdomen. Patients were satisfied with the needle size (76% completely satisfied), and 93% reported never deliberately missing a dose.

### OPAQ‐SV

According to the OPAQ‐SV, patients were generally in good health (physical function: 86 ± 16), although a considerable proportion of patients reported suboptimal emotional health (74 ± 18) and back pain (70 ± 22).

### EQ‐5D‐5Land VAS

Most patients rated their overall health as good per EQ‐5D‐5L (mean = 0.80; median = 0.90; SD = 0.21) and EQ‐5D‐VAS (mean = 75.8, median = 80.0, SD = 17.14).

### Patient perspective of disease and its management

The majority of patients (80%) reported being “very” or “extremely” knowledgeable about their osteoporosis treatment options. The majority (69%) noted that their healthcare team understands how osteoporosis impacts their lives. Fifty‐five percent of patients discussed treatment options in detail with their healthcare team, and 27% frequently asked their healthcare team questions regarding treatment choices.

### Subgroup analyses

Anabolic‐naïve patients (cohort I) had a higher level of satisfaction with ABL than anabolic switch patients (cohort II). In particular, anabolic switchers were less satisfied with the injection site, needle size, and having to inject the medication overall. Despite these differences, the average adherence score (mean ± SD) was comparable for patients in cohort I (7.0 ± 1.1) versus cohort II (7.1 ± 1.2). In a subgroup analysis of anabolic switchers (*n* = 43), the median time taken to switch was 12 months (IQR 6, 18). The average number of days (mean ± SD) between TPTD discontinuation and ABL initiation for those who switched due to lack of treatment efficacy (*n* = 4) was (809 ± 592.1) versus those who switched for other reasons (651.7 ± 1208.4). Median time from TPTD initiation to ABL initiation was 1 year (IQR 0.5, 1.5).

### Multivariable analyses

#### Predictors of treatment satisfaction

In logistic regression analyses, patients who found ABL easy to prepare (OR = 2.62; 95% CI, 1.01–6.81; *p* = 0.048) and those who had a higher number of fractures in the year prior to treatment initiation (OR = 1.72; 95% CI, 1.03–2.86; *p* = 0.037) were significantly more likely to have a favorable satisfaction score (measured by a single question, “overall how satisfied are you with abaloparatide” and dichotomized as completely satisfied [score of 5] or not completely satisfied [score of 1, 2, 3, 4]).

Ease of remembering to take medication was a significant predictor of treatment satisfaction (using TSQM Global Satisfaction score) (coefficient [SE]: 7.97 [3.81]; 95% CI, 0.45–15.49; *p* = 0.038) (Table 3). In addition, those who found medication easy to prepare had higher satisfaction with treatment convenience (coefficient [SE]: 13.60 [2.79]; 95% CI, 8.08–19.12; *p* < 0.0001) after adjusting for potential confounders, including but not limited to, treatment history and co‐pay (Table 4).

**Table 3:** Predictors of Satisfaction (TSQM Global Satisfaction Score) (*n* = 161) (Multivariable Regression Tables)

Variable	Coefficient (SE)	*t*	*p* > |*t*|	95% CI
Prior anabolic treatment				
Anabolic naïve	0‐base			
Anabolic experienced	5.01 (3.27)	1.53	0.128	−1.45, 11.48
Copay	0.0018 (0.01)	0.45	0.654	−0.01, 0.01
FRAX				
Low risk	0‐base			
High risk	−7.67 (3.30)	−2.33	0.021	−14.20, −1.15
Prepare	5.65 (3.90)	1.45	0.150	−2.06, 13.36
Recall	7.97 (3.81)	2.09	0.038	0.45, 15.49
Travel	0.52 (2.35)	0.22	0.826	−4.13, 5.16
Age	0.12 (0.19)	0.63	0.527	−0.25, 0.48
Understanding	6.15 (3.26)	1.88	0.061	−0.30, 12.60
Support				
No	0‐base			−11.01, 31.73
Yes	10.36 (10.82)	0.96	0.340	
Cons	−36.66 (26.37)	−1.39	0.17	−88.77, 15.45

0‐base = base or reference category; Cons = estimate of baseline odds; Copay = average out‐of‐pocket cost for abaloparatide monthly; FRAX = Fracture Risk Assessment Tool; Prepare = whether it is easy to prepare abaloparatide; Recall = whether it is easy to recall to take abaloparatide; Support = whether the patient feels that he/she has enough support on how to take abaloparatide; Travel = whether it is easy to travel with abaloparatide; TSQM Global = treatment satisfaction questionnaire measure for medication global satisfaction domain; Understanding = whether the patient's doctor/team explained how to use abaloparatide.

**Table 4:** Predictors of Satisfaction With Treatment Convenience (TSQM Convenience Score) (*n* = 163) (Multivariable Regression Tables)

Variable	Coefficient (SE)	*t*	*p* > |*t*|	95% CI
Prior anabolic treatment				
Anabolic naïve	0‐base			
Anabolic experienced	−0.006 (2.33)	−0.00	0.998	−4.62, 4.60
Copay	−0.005 (0.003)	−1.78	0.076	−0.011, 0.00
FRAX				
Low risk	0‐base			
High risk	−1.34 (2.35)	−0.57	0.568	−5.98, 3.30
Prepare	13.60 (2.79)	4.87	0.000	8.08, 19.12
Recall	4.44 (2.73)	1.63	0.106	−0.95, 9.84
Travel	3.24 (1.69)	1.92	0.057	−0.10, 6.57
Age	−0.068 (0.13)	−0.51	0.611	−0.33, 0.19
Understanding	−1.12 (2.35)	−0.48	0.634	−5.76, 3.52
Support				
No	0‐base			
Yes	12.24 (7.78)	1.57	0.118	−3.13, 27.61
Cons	−17.71 (18.91)	−0.94	0.351	−55.06, 19.65

0‐base = base or reference category; Cons = estimate of baseline odds; Copay = average out of pocket cost for abaloparatide monthly; FRAX = Fracture Risk Assessment Tool; Prepare = whether it is easy to prepare abaloparatide; Recall = whether it is easy to recall to take abaloparatide; Support = whether the patient feels that he/she has enough support on how to take abaloparatide; Travel = whether it is easy to travel with abaloparatide; TSQM Convenience = treatment satisfaction questionnaire measure for medication convenience domain; Understanding = whether the patient's doctor/team explained how to use abaloparatide.

Fracture risk as measured by the modified FRAX was negatively associated with overall satisfaction score (coefficient [SE]: −7.67 [3.30]; 95% CI, −14.20 to −1.15; *p* = 0.021) (Table 3) and with satisfaction with treatment effectiveness (coefficient [SE]: −8.19 [3.27]; 95% CI, −14.66 to −1.73; *p* = 0.013). Fracture risk as measured by prior fracture history, however, was not a significant predictor of TSQM Global Satisfaction score (coefficient [SE]: 0.13 [1.41]; 95% CI, −2.67 to 2.92; *p* = 0.93), satisfaction with treatment effectiveness (coefficient [SE]: 2.45 [1.40]; 95% CI, −0.31 to 5.22; *p* = 0.08), or satisfaction with treatment convenience (coefficient [SE]: 0.61 [0.99]; 95% CI, −1.35 to 2.56; *p* = 0.54).

#### Predictors of ease of use

Several models evaluated “ease of use” as measured by “storage,” “preparation,” “remembering to take medication,” and “travel.” Patients who were satisfied with ease of medication preparation (coefficient [SE]: 0.33 [0.9]; 95% CI, 0.16–0.50; *p* < 0.0001), ease of travel (coefficient [SE]: 0.21 [0.05]; 95% CI, 0.11–0.31; *p* < 0.0001), and frequency of medication intake (coefficient [SE]: 0.12 [0.04]; 95% CI, 0.05–0.19, *p* < 0.0001) were also significantly more likely to consider ABL easy to store (Table 5).

**Table 5:** Predictors of Treatment Ease of Use (*n* = 158) (Multivariable Regression Tables)

Variable	Coefficient (SE)	*t*	*p* > |*t*|	95% CI
OP support group				
No	0‐base			
Yes	0.03 (0.06)	0.40	0.693	−0.10, 0.15
FRAX				
Low risk	0‐base			
High risk	0.08 (0.063)	1.29	0.201	−0.04, 0.20
Age	−0.004 (0.0034)	−1.08	0.282	−0.01, 0.003
BMI	−0.0008304 (0.006)	−0.15	0.885	−0.012, 0.01
Rheumatoid arthritis				
No	0‐base			
Yes	0.043 (0.13)	0.32	0.747	−0.22, 0.31
Where	0.02 (0.06)	0.35	0.728	−0.09, 0.13
Often	0.12 (0.04)	3.42	0.001	0.05, 0.19
Size	−0.005 (0.06)	−0.09	0.931	−0.12, 0.11
Prepare	0.33 (0.09)	3.85	0.000	0.16, 0.50
Recall	−0.01(0.08)	−0.019	0.850	−0.17, 0.14
Travel	0.21 (0.05)	4.12	0.000	0.11, 0.31
Understanding	−0.07 (0.07)	−1.11	0.269	−0.21, 0.06
Support				
No	0‐base			
Yes	0.04 (0.24)	0.18	0.858	−0.43, 0.52
Cons	2.28 (0.59)	3.88	0.000	1.12, 3.43

0‐base = base or reference category; BMI = body mass index; Cons = estimate of baseline odds; FRAX = Fracture Risk Assessment Tool; Often = how often patient injects medication; OP support = whether the patient is taking part in support groups to manage osteoporosis; Prepare = whether it is easy to prepare abaloparatide; RA = rheumatoid arthritis as a concomitant condition; Recall = whether it is easy to recall to take abaloparatide; Size = size of the needle used to inject abaloparatide; Support = whether the patient feels that he/she has enough support on how to take abaloparatide; Travel = whether it is easy to travel with abaloparatide; Understanding = whether the patient's doctor/team explained how to use abaloparatide; Where = part of the body patient injects abaloparatide.

Patients who were satisfied with the site of injection (coefficient = 0.15; 95% CI, 0.053–0.26; *p* = 0.003) and medication storage (coefficient = 0.28; 95% CI, 0.14–0.43; *p* < 0.0001) were also more satisfied with ease of preparation. Having rheumatoid arthritis as a comorbidity was significantly associated with remembering to take ABL (coefficient = 0.30; 95% CI, 0.02–0.58; *p* = 0.036).

#### Predictors of adherence

Ease of remembering to take ABL was a significant predictor of self‐reported adherence (OR = 16.66; 95% CI, 3.30–84.24; *p* = 0.001). Although “having to inject the medicine” was considered a bothersome attribute of treatment by study participants, it was not a significant predictor of self‐reported adherence.

## Discussion

### Summary

This is the first ABL patient experience study, and it indicates that the majority of patients were satisfied with treatment and found it to be convenient and easy to prepare and store. The positive experience with ABL and high self‐reported adherence to therapy may reflect a high level of support provided by the prescriber and opportunities for shared decision making from the participating sites for the study including patients' understanding of disease and treatment. Ability to build bone, reduce fracture risk, and allow for daily activities were most frequently reported as favorable features.

Patients who found ABL easy to prepare and convenient to use were significantly more likely to be satisfied with treatment. Ease of recall to take medication was also a predictor of higher self‐reported adherence. Although having to inject the medicine and out‐of‐pocket costs were considered the worst treatment attributes, they were not significant predictors of treatment adherence, indicating patients' willingness to take treatment despite the mode of intake. Patient self‐perception of benefit/risk may have played a role in treatment satisfaction and corresponding higher adherence with therapy because those with a higher number of prior fractures, who may have considered themselves at high risk, were also more likely to report higher adherence and more likely to be satisfied with treatment effectiveness.

Persistence with osteoporosis medication in the real world is generally low, varying between 29% to 68% at 1‐year after treatment initiation for various therapies.^(^ ^23^ ^)^ Reasons for discontinuation include concerns about side effects, perceived lack of efficacy, difficulty taking medication as directed, as well as medication expenses.^(^ ^24^ ^)^ Suboptimal persistence reduces treatment effectiveness and ultimately increases risk of subsequent fractures by as much as 40%.^(^ ^25^ ^)^ Patient perspective of treatment and benefit/risk assessment may be different from that of the healthcare provider and may contribute to patient attitude toward therapy and medication‐taking behavior. As such, it is important to assess patient perspective to identify issues with therapy that could be addressed to improve adherence and to support the value proposition of newly approved treatments. In fact, consideration of patient perspective is increasingly important in evaluation of new products as noted by the FDA's Patient‐Focused Drug Development Initiative^(^ ^26^ ^)^ and by the Institute for Clinical and Economic Review (ICER) 2020 value assessment framework.^(^ ^27^ ^)^

### Study limitations

The results should be interpreted within the context of study limitations. First, selection bias may have been introduced due to the nonrandom selection of sites participating in the study, although efforts were made to identify sites representative of specific settings. To minimize this bias, each participating site was asked to recruit eligible patients using a systematic approach, eg, by inviting patients into the study on a consecutive basis until the target sample was met. In addition, there could be bias due to the selection of study participants. Data on acceptance for participation were not collected. None of the participants who enrolled in the study had previously used ABL.

Second, generalizability of study results may be limited by the use of a study population based on a convenience sample of patients; hence, the patients and treatment patterns observed in this study may not reflect all real‐world ABL patients. The majority of patients had some college degree (58%), with only 5% unemployed or on disability allowance. The data on lifestyle risk factors (ie, alcohol intake, smoking, and physical activity level) were reflective of a healthier population of patients. It is therefore likely that our patient population includes those with a higher socioeconomic status, which could be associated with better access to healthcare services and/or treatment and thus better perception of care. Third, study data, generated from patient medical charts, are subject to missing data due to variance in recorded information by practice and medical staff. To overcome this issue, the study used standardized eCRF, which went through a thorough review process prior to implementation to help ensure that the content of the eCRF was appropriate for the present study objectives and use in a real‐world setting. In addition, a data management plan was implemented to ensure quality control. Data regarding lifestyle factors and adherence are self‐reported and subject to underreporting bias.

We did not have all of the variables required for FRAX scoring nor complete data for all study participants, including data on fracture risk associated with lifestyle factors. We did, however, calculate modified FRAX score for 99% (191/193) of participants. We also did not ascertain specific data pertaining to full medical history. Although some disease‐specific instruments were used to assess patient experience with ABL (eg, OPAQ, OS‐MMAS), where these measures were not available, a general instrument was used instead (eg, TSQM‐9, EQ‐5D‐5L, and VAS). It is important to note that although the side effects domain was not included in TSQM‐9, any unpleasant experiences with a medication were likely to be captured in the TSQM global satisfaction items. As a result, even without the side effects items, the TSQM‐9 allows for patients to weigh the pros and cons of medication and the less‐favorable aspects of patients' experiences with their medications would be captured.

Given the small number of patients with additional *T* scores, posttreatment initiation, and the variability in timing of assessment, evaluation of change in BMD *T* scores was out of the scope of the current study and will be assessed in the follow‐up evaluation. Furthermore, BTMs were not collected for the majority of patients, and evaluation of changes from baseline to follow‐up will also be addressed in the longitudinal study of outcomes. Last, approximately half of patients had only been taking ABL for 6 months or less, and treatment adherence may change over a longer follow‐up time. Additional studies are required to evaluate predictors of early versus late treatment discontinuation.

In conclusion, this real‐world study for the first time provides data on patient experience with ABL and supports a favorable perception of therapy in terms of treatment satisfaction, ease of use, and adherence. The study also highlights the importance of shared decision making and access to adequate support from the healthcare team in patient attitude toward treatment. Future research on treatment outcomes, given availability of longitudinal data, are warranted to further support the value proposition of ABL in patients at high risk for fracture.

## Disclosures

DTG is a consultant for Eli Lilly and Co. (Eli Lilly) and Radius Health, Inc. RW, YW, and SAW are employees of and own company stock in Radius Health, Inc. TB is an investigator for Radius Health, Inc. and her institution received funding to conduct the study reported here; she reports being a former speaker for Radius Health, Inc., Amgen, and Eli Lilly. CD has participated in advisory boards and is a speaker for Amgen and Eli Lilly and is a speaker for Radius Health, Inc. RSE is a consultant for Radius Health, Inc. ALJ is a speaker for Amgen. JMK is a consultant for Radius Health, Inc., a speaker for Amgen and Radius Health, Inc., and has participated in advisory boards for Amgen and Radius Health, Inc. MM has no conflicts of interest to disclose. MS is a speaker/has participated in advisory boards for Abbvie, Eli Lilly, Pfizer, Radius Health, Inc., and UCB. TV is a speaker, advisor, and investigator for Radius Health, Inc., advisor and investigator for Takeda, and investigator for Ascendis. JR and TomB are employees of Adelphi Real World, Bollington, UK, and are paid consultants of Radius Health, Inc.

## AUTHOR CONTRIBUTIONS

**Deborah Gold:** Conceptualization; methodology; visualization; writing‐review and editing. **Rich Weiss:** Conceptualization; methodology; visualization; writing‐review and editing. **Tammy Beckett:** Investigation; resources; writing‐review and editing. **Chad Deal:** Investigation; resources; writing‐review and editing. **Robert Epstein:** Conceptualization; investigation; resources; writing‐review and editing. **Andrew James:** Investigation; resources; writing‐review and editing. **Jacqueline Kernaghan:** Investigation; resources; writing‐review and editing. **Mahshid Mohseni:** Investigation; resources; writing‐review and editing. **Michael Spiegel:** Investigation; resources; writing‐review and editing. **Tamara Vokes:** Investigation; resources; writing‐review and editing. **Jenna Roberts:** Data curation; formal analysis; project administration; software; validation; visualization; writing‐review and editing. **Tom Bailey:** Data curation; formal analysis; project administration; software; validation; visualization; writing‐review and editing. **Yamei Wang:** Conceptualization; formal analysis; writing‐review and editing. **Setareh Williams:** Conceptualization; funding acquisition; methodology; resources; supervision; visualization; writing‐original draft; writing‐review and editing.

## Supporting information


**Supplemental Item 1.** Patient questionnaire (PDF)
**Supplemental Item 2.** Electronic Case Report Form (eCRF) (PDF)
