# Supplementary material for: Abaloparatide Real‐World Patient Experience Study
Source: JBMR Plus. 2021 Feb 4;5(3):e10457. doi: 10.1002/jbm4.10457 (PMC7990148; doi:10.1002/jbm4.10457)
Supplement: Supplementary file 1 — Supplemental Item 1. Patient questionnaire (PDF) Supplemental Item 2. Electronic Case Report Form (eCRF) (PDF) [file JBM4-5-e10457-s001.pdf]

Supplementary Material

Supplementary Item 1: Patient questionnaire (PDF)

Supplementary Item 2: Electronic Case Report Form (eCRF) (PDF)

Patient Questionnaire

Site ID	Patient number
**Do not write initials, names or any other types of personal identification anywhere on this form**	

A. YOUR OSTEOPOROSIS TREATMENT

Q1. For each of the following, please say how satisfied you are with TYMLOS (abaloparatide)? Please ✓ a rating per row, from 1 (not at all satisfied) to 5 (completely satisfied)

	1 Not at all satisfied	2	3	4	5 Completely satisfied	Unsure
Whether the medication allows you to do daily activities	☐	☐	☐	☐	☐	☐
Whether the medication helps build bone	☐	☐	☐	☐	☐	☐
Whether the medication helps reduce fracture risk	☐	☐	☐	☐	☐	☐
How taking the medication fits with your day to day schedule	☐	☐	☐	☐	☐	☐
How often you have to take the medication	☐	☐	☐	☐	☐	☐
Whether it is easy to prepare the medication	☐	☐	☐	☐	☐	☐
Whether it is easy to store the medication	☐	☐	☐	☐	☐	☐
Whether it is easy to travel with the medication	☐	☐	☐	☐	☐	☐
Whether it is easy to remember to take the medication	☐	☐	☐	☐	☐	☐
How much you pay out of your own money for the medication	☐	☐	☐	☐	☐	☐
Having to inject the medication	☐	☐	☐	☐	☐	☐
The size of the needle used to inject your medication	☐	☐	☐	☐	☐	☐
The part of your body (stomach) where you need to inject your medication	☐	☐	☐	☐	☐	☐

Q2. For each of the following, please select a) the single *best feature* and b) the single *most bothersome* feature you experience with TYMLOS (abaloparatide)? Please ✓ **<u>one</u>** feature per column

	Q2a. Best feature	Q2b. Most bothersome feature
Whether the medication allows you to do daily activities	☐	☐
Whether the medication helps build bone	☐	☐
Whether the medication helps reduce fracture risk	☐	☐
How taking the medication fits with your day to day schedule	☐	☐
How often you have to take the medication	☐	☐
Whether it is easy to prepare the medication	☐	☐
Whether it is easy to store the medication	☐	☐
Whether it is easy to travel with the medication	☐	☐
Whether it is easy to remember to take the medication	☐	☐
How much you pay out of your own money for the medication	☐	☐
Having to inject the medication	☐	☐
The size of the needle used to inject your medication	☐	☐
The part of your body stomach) where you need to inject your medication	☐	☐

A. YOUR OSTEOPOROSIS TREATMENT (continued)

Q3. TYMLOS (abaloparatide) should be injected in your lower abdomen. In which part of your body, do you usually inject your TYMLOS (abaloparatide) medication?

☐ Lower Abdomen ☐ Thigh ☐ Other

Q4. TYMLOS comes in the form of an injectable pen that should be used over a 30 day period. Do you ever deliberately try to make your pen last longer than this by missing doses and using beyond 30 days?

☐ Often ☐ Sometimes ☐ Never

Q5. Overall how satisfied are you with TYMLOS (abaloparatide) medication?
Please select a rating below, from 1 (not at all satisfied) to 5 (completely)

1	2	3	4	5
☐ Not at all satisfied	☐	☐	☐	☐ Completely satisfied

Q6a. When you go to collect your TYMLOS (abaloparatide) medication, have you ever had a delay between handing in your prescription and getting your medication that was longer than one day?

☐ Yes ☐ No → Go to Q7

Q6b. Thinking about the last time this happened, how long was the delay?

__________ days ☐ Unsure

Q6c. Thinking about the last time this happened, do you know what caused the delay?

☐ An incomplete prescription ☐ Medication out of stock
☐ Other/ unsure

Q7. When you started treatment with TYMLOS (abaloparatide) overall, how well did your doctor (or other member of your healthcare team) explain how to use the medication?
Please select a rating from 1 (not very well explained) to 5 (very well explained)

1	2	3	4	5
☐ Not very well explained	☐	☐	☐	☐ Very well explained

Q8. Since starting TYMLOS (abaloparatide), do you feel that you have enough support with how to take this medication?

☐ Yes ☐ No ☐ Don’t know

A. YOUR OSTEOPOROSIS TREATMENT (continued)

Q9. What time of day do you normally take your TYMLOS (abaloparatide) medication?

☐ Morning ☐ Afternoon ☐ Evening ☐ Night ☐ It varies

Q10. How important is it to you that your osteoporosis medication is convenient to store?
Please select a rating from 1 (not very important) to 5 (very important)

1	2	3	4	5
☐ Not very important	☐	☐	☐	☐ Very important

Q11. How easy/ convenient is it to store your TYMLOS medication?
Please select a rating from 1 (very easy) to 5 (very difficult)

1	2	3	4	5
☐ Not very easy/ convenient	☐	☐	☐	☐ Very easy/ convenient

Q12a. In an average month, how much do you pay out of your own money (excluding any money you may receive to support your disease, e.g. from your government) for the below expenses, in relation to your osteoporosis condition?

Please write "$0" if you spend nothing on any of these items.

	Approximate cost ($ monthly)
Daily assistance (i.e. from a nurse or paid caregiver)	$________
Osteoporosis medications, **<u>excluding TYMLOS (abaloparatide)</u>**. This includes both prescription and non-prescription medication	$________
Travel to doctor/hospital appointments	$________
Tests, i.e. blood tests, scans, etc.	$________
Parking fees (i.e. at the pharmacy, or hospital/clinic)	$________
Other costs incurred due to your osteoporosis	$________

Q12b. In an average month, how much do you pay out of your own money (excluding any money you may receive to support your disease, i.e. from your government) specifically for your TYMLOS (abaloparatide) medication?

Approximate cost
($ monthly)

$________

A. YOUR OSTEOPOROSIS TREATMENT

Q13a. In your opinion, do you think that there was an unnecessary delay between being diagnosed with osteoporosis and being prescribed your first drug treatment?

☐ Yes ☐ No → Go to Q14

Q13b. In your own opinion, why do you think this was?
(please ✓ all that apply)

- ☐ My doctor wanted to wait and see if my symptoms got worse
- ☐ I needed to see a specialist before medication could be prescribed
- ☐ I needed to wait for test results before I could be prescribed a medication
- ☐ My health insurance would not cover the medication my doctor wanted to prescribe
- ☐ Other

If you used to take a different medication for your osteoporosis before starting TYMLOS (abaloparatide) please answer question 14 thinking about the last treatment or treatment combination that you took for your osteoporosis.

If you have never taken any medication for osteoporosis, please skip to the next page.

Q14. Overall, how satisfied were you with <u>your last treatment</u> for your osteoporosis?
Please select a rating below, from 1 (not at all satisfied) to 5 (completely)

1	2	3	4	5
☐ Not at all satisfied	☐	☐	☐	☐ Completely satisfied

PLEASE CONTINUE TO THE NEXT PAGE

INSERT TSQM

TSQM Webpage: https://www.iqvia.com/landing/treatmentsatisfaction-questionnaire-for-medication-tsqm

B. MEDICATION ADHERENCE

Osteoporosis-Specific Morisky Medication Adherence Scale (©OS-MMAS)

You indicated that you are taking medication for your osteoporosis. Individuals have identified several issues regarding their medication-taking behavior and we are interested in your experiences. There is no right or wrong answer. Please answer each question based on your personal experience with your osteoporosis medication (ie, other than calcium and vitamin D)

(Please check your response below)

	No=1	Yes=0
1. Do you sometimes forget to take your osteoporosis medication (ie, other than calcium and vitamin D)?		
2. People sometimes miss taking their medications for reasons other than forgetting. Thinking over the past 4 weeks, were there any days when you did not take your osteoporosis medication?		
3. Have you ever cut back or stopped taking your osteoporosis medication without telling your doctor, because you felt worse when you took it?		
4. When you travel or leave home, do you sometimes forget to bring along your osteoporosis medication?		
5. Did you take your osteoporosis medication the last time you were supposed to take it?		
6. If you feel that your osteoporosis medication is not working, do you sometimes stop taking your medication?		
7. Taking medication exactly as prescribed is a real inconvenience for some people. Do you ever feel hassled about sticking to your osteoporosis treatment plan?		

8. How often do you have difficulty remembering to take all your medications?

(**Please circle your response below)**

Never/Rarely…………………………………….....**4**

Once in a while……………………………….…**3**

Sometimes……………………………………….....**2**

Usually…………………………………………..…**1**

All the time……………………………………..….**0**

Use of the ©OS-MMAS is protected by US copyright laws. Permission for use is required. Licensure agreement is available from: Donald E. Morisky ScD ScM MSPH, Professor, Department of Community Health Sciences, UCLA School of Public Health, 650 Charles E. Young Drive South, Los Angeles, CA 90095-1772. ©Morisky 2009 OS-MMAS, US English Version 1.0

C. YOUR DAY TO DAY FUNCTIONING (OPAQ-SV)

THE OSTEOPOROSIS ASSESSMENT QUESTIONNAIRE Short Version (OPAQ-SV)

Copyright. Mapi All rights reserved

Please answer the following questions about your health. Most questions ask about your health during the past two weeks. There are no right or wrong answers to the questions. It is very important that you answer every question

WALKING/BENDING

How often were you able to do daily shopping or errands?

☐ All days ☐ Most days ☐ Some days ☐ Few days ☐ No days

How often were you in bed or chair for most of the day?

☐ All days ☐ Most days ☐ Some days ☐ Few days ☐ No days

How often were you able to do sports and games that you would like to do?

☐ All days ☐ Most days ☐ Some days ☐ Few days ☐ No days

How often were you able to do walk as much as you needed to do?

☐ All days ☐ Most days ☐ Some days ☐ Few days ☐ No days

How often did you have trouble bending, lifting or stooping?

☐ All days ☐ Most days ☐ Some days ☐ Few days ☐ No days

How often did you have trouble walking a block or climbing one flight of stairs?

☐ All days ☐ Most days ☐ Some days ☐ Few days ☐ No days

How often did you need to use a cane, crutches, walker, or companion while walking?

☐ All days ☐ Most days ☐ Some days ☐ Few days ☐ No days

C. YOUR DAY TO DAY FUNCTIONING OPAQ-SV (continued)

DAILY ACTIVITIES

Could you easily put on or take off a pair of stockings and/or underwear?

☐ All days ☐ Most days ☐ Some days ☐ Few days ☐ No days

Could you easily comb, brush, or style your hair?

☐ All days ☐ Most days ☐ Some days ☐ Few days ☐ No days

Could you easily reach shelves that were above your head?

☐ All days ☐ Most days ☐ Some days ☐ Few days ☐ No days

Have you had to change the way you bathe yourself?

☐ Always ☐ Very often ☐ Sometimes ☐ Almost never ☐ Never

How you had to change the type of clothes you wear because of difficulty in dressing?

☐ Always ☐ Very often ☐ Sometimes ☐ Almost never ☐ Never

How often were you able to do light housework such as cooking without help?

☐ Always ☐ Very often ☐ Sometimes ☐ Almost never ☐ Never

How often were you able to do heavy housework such as vacuuming without help?

☐ Always ☐ Very often ☐ Sometimes ☐ Almost never ☐ Never

How often were able to do your daily work, either at home, as a volunteer, at school, or at a paid job?

☐ Always ☐ Very often ☐ Sometimes ☐ Almost never ☐ Never

C. YOUR DAY TO DAY FUNCTIONING OPAQ-SV (continued)

TRANSFER

How often do you have trouble getting in or out of bed?

☐ All days ☐ Most days ☐ Some days ☐ Few days ☐ No days

How often do you have trouble getting in or out of a chair?

☐ All days ☐ Most days ☐ Some days ☐ Few days ☐ No days

How often do you have trouble getting in or out of the toilet?

☐ All days ☐ Most days ☐ Some days ☐ Few days ☐ No days

How often do you have trouble getting in or out of cars or public transportation?

☐ All days ☐ Most days ☐ Some days ☐ Few days ☐ No days

FEAR OF FALLS

How often were you afraid that you would fall?

☐ Always ☐ Very often ☐ Sometimes ☐ Almost never ☐ Never

How often were you afraid that you would accidentally break or fracture a bone?

☐ Always ☐ Very often ☐ Sometimes ☐ Almost never ☐ Never

How often did you feel that you were losing balance?

☐ Always ☐ Very often ☐ Sometimes ☐ Almost never ☐ Never

How often did you use a hand rail or other support when walking up or down stairs?

☐ Always ☐ Very often ☐ Sometimes ☐ Almost never ☐ Never

How often did your fear of falling keep you from doing what you want to do?

☐ Always ☐ Very often ☐ Sometimes ☐ Almost never ☐ Never

C. YOUR DAY TO DAY FUNCTIONING OPAQ-SV (continued)

BACK PAIN

How often did you have any back ache or pain?

☐ All days ☐ Most days ☐ Some days ☐ Few days ☐ No days

How would you describe the back ache or pain you usually had?

☐ Severe ☐ Moderate ☐ Mild ☐ Very mild ☐ None ☐ I had no back pain

How often did your back feel stiff for more than one hour from the time you woke up?

☐ All days ☐ Most days ☐ Some days ☐ Few days ☐ No days

How often did back ache or pain keep you from doing what you wanted to do?

☐ All days ☐ Most days ☐ Some days ☐ Few days ☐ No days

BODY IMAGE

How often were you aware of changes in your body when trying on clothes?

☐ Always ☐ Very often ☐ Sometimes ☐ Almost never ☐ Never

How often were you bothered by the way your back looks?

☐ Always ☐ Very often ☐ Sometimes ☐ Almost never ☐ Never

How often were you concerned by changes in the way your body looks?

☐ Always ☐ Very often ☐ Sometimes ☐ Almost never ☐ Never

INDEPENDENCE

How often did you feel confident you could live on your own without assistance?

☐ Always ☐ Very often ☐ Sometimes ☐ Almost never ☐ Never

How often did you have to rely on others for assistance of daily activities?

☐ Always ☐ Very often ☐ Sometimes ☐ Almost never ☐ Never

How often were you worried that you might not be able to take care of yourself in the future?

☐ Always ☐ Very often ☐ Sometimes ☐ Almost never ☐ Never

D. MANAGEMENT OF YOUR OSTEOPOROSIS

Q1. Do you feel you know enough about your osteoporosis treatment options?

☐ Not at all ☐ Slightly ☐ Moderately ☐ Very ☐ Extremely

Q2. How involved are you in decisions about your osteoporosis treatment?

☐ I do not get involved at all ☐ Occasionally I ask questions ☐ I frequently ask questions ☐ I discuss my treatment choices in detail

Q3a. Do you receive additional support through any educational programs or support groups/ networks for your osteoporosis?

☐ Yes, and I find it useful to help me manage my disease
☐ Yes, but it currently has limited impact on helping me to manage my disease
☐ No, but I would like to become a member to help me manage my disease
☐ No, and I currently have no interest in becoming a member

Q3b. If you have questions about your osteoporosis in between routine visits to the doctor, is there a healthcare professional (e.g. a nurse) that you can easily get in touch with?

☐ Yes ☐ No

Q4. How has osteoporosis affected your life? *(please ✓ <u>all that apply</u>)*

☐ It has not affected my lifestyle
☐ I am less active
☐ I've given up personal hobbies
☐ Increased my fear of falls
☐ I've cut back on my social life with family and friends
☐ I have to rely on family / caregivers more
☐ It has increased my financial burden
☐ Increased my fear of losing independence
☐ Other ways

Q5a. Have you taken part in any of the following educational programs to help you manage your osteoporosis?
(please ✓ <u>all that apply</u>)

☐ Nutritional support/ (i.e. advice about what you should eat)
☐ Exercise programs
☐ Disease education (i.e. programs to teach you more about living with osteoporosis)
☐ Drug/ treatment education (i.e. programs to teach you about your medication)
☐ Fall prevention programs (i.e. advice to help you avoid falling over)
☐ Other
☐ None

D. MANAGEMENT OF YOUR OSTEOPOROSIS (continued)

Q5a. Have you been involved with any of the following support groups for your osteoporosis?
(please ✓ all that apply)

- ☐ Live support groups where you see people in person
- ☐ Social media or online support groups
- ☐ Financial assistance/ insurance support for treatment
- ☐ None

Q6. Has your doctor or other member of your health care team ever spoken to you about test results that show how well your osteoporosis medication is working?

☐ Yes ☐ No ☐ Unsure

Q7. Based on your experience with your main doctor and other members of the healthcare team who treat your osteoporosis, to what extent do you agree with the following statements with regards to your osteoporosis condition?
Please rate each statement below, where 1 (strongly disagree); to 7 (strongly agree)

	1 Strongly disagree	2	3	4	5	6	7 Strongly agree
My healthcare team listens to my concerns and addresses my questions	☐	☐	☐	☐	☐	☐	☐
My healthcare team keeps me informed about new treatment options	☐	☐	☐	☐	☐	☐	☐
My healthcare team understands how much my condition impacts my life	☐	☐	☐	☐	☐	☐	☐
I feel comfortable discussing things with my healthcare team	☐	☐	☐	☐	☐	☐	☐
I am confident that I am getting the best treatment from my healthcare team	☐	☐	☐	☐	☐	☐	☐
My healthcare team and I have similar goals for my osteoporosis management and/or treatment	☐	☐	☐	☐	☐	☐	☐
My healthcare team is aware of my concerns (if any) about my treatments	☐	☐	☐	☐	☐	☐	☐

E. YOUR CURRENT HEALTH STATE

Q1. Compared to other illnesses or health concerns you may have, how much of a priority is your osteoporosis?

Please circle a rating from 1 (osteoporosis is the lowest priority relative to other health concerns) to 10 (osteoporosis is the highest priority relative to other health concerns)

1	2	3	4	5	6	7	8	9	10
Lowest priority				Moderate priority					Highest priority

Q2. How would you rate the level of your pain over the last 24 hours (in relation to your osteoporosis)?

Please circle one number on the scale, where 1 means "no pain" and 10 means "worst pain"

1	2	3	4	5	6	7	8	9	10
**No pain**				**Moderately painful**					**Worst pain**

Q3. How often are you physically active (for e.g., walking, gardening, brisk pushing a stroller, or climbing the stairs)?

☐ Not active at all ☐ Once a week ☐ 2 or 3 times per week ☐ 4 or more times per week

Q4. Over the past month, how many times have you fallen over?

________ times OR ☐ I have not fallen over in the last month

PLEASE CONTINUE TO THE NEXT PAGE

E. YOUR CURRENT HEALTH STATE (continued)

PLEASE COMPLETE THE QUESTIONS BELOW AND THE EXERCISE ON THE NEXT PAGE

By *placing a check* in one box in each group below, please indicate which statements best describe your own health today

1. Mobility

I have no problems in walking about ☐

I have some problems in walking about ☐

I am confined to bed ☐

2. Self-Care

I have no problems with self-care ☐

I have some problems washing or dressing myself ☐

I am unable to wash or dress myself ☐

3. Usual Activities (e.g. *work, study, housework, family or leisure activities*)

I have no problems with performing my usual activities ☐

I have some problems with performing my usual activities ☐

I am unable to perform my usual activities ☐

4. Pain/Discomfort

I have no pain or discomfort ☐

I have moderate pain or discomfort ☐

I have extreme pain or discomfort ☐

5. Anxiety/Depression

I am not anxious or depressed ☐

I am moderately anxious or depressed ☐

I am extremely anxious or depressed ☐

© 1990 EuroQol Group EQ-5D™ is a trade mark of the EuroQol Group

E. YOUR CURRENT HEALTH STATE (continued)

To help people say how good or bad a health state is, we have drawn a scale (rather like a thermometer) on which the best state you can imagine is marked 100 and the worst state you can imagine is marked 0.

We would like you to indicate on this scale how good or bad your own health is today, in your opinion. Please do this by drawing a line from the box below to whichever point on the scale indicates how good or bad your health state is today

Your own health state today

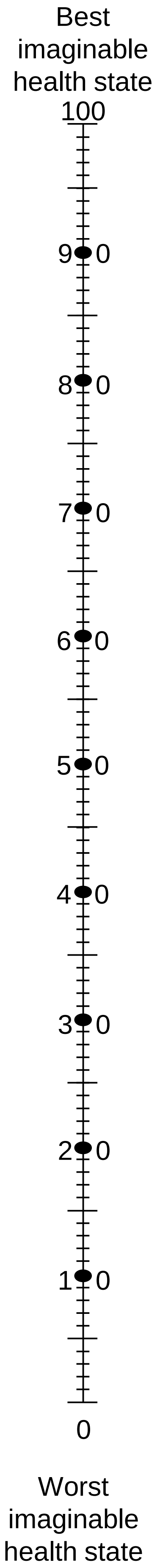

© 1990 EuroQol Group EQ-5D™ is a trade mark of the EuroQol Group

SECTION F: ABOUT YOU

Q1. What is your age? ________ years

Q2. Level of education? (Please ✓ one box only)

- ☐ Less than high school
- ☐ High school diploma or GED
- ☐ College Degree (2 yr - Associates)
- ☐ College Degree (4 yr - Bachelor)
- ☐ Graduate Degree or higher
- ☐ Trade School/certificate program
- ☐ Other

Q3. Current employment status?

- ☐ Working full time
- ☐ Working part time
- ☐ On long term sick leave
- ☐ Homemaker
- ☐ Student
- ☐ Retired
- ☐ Unemployed
- ☐ Don't know

Q4. Current total household income each year? *(Please ✓ one box only)*

- ☐ Less than $5,000
- ☐ $5,001 - $15,000
- ☐ $15,001 - $25,000
- ☐ $25,001 - $50,000
- ☐ $50,001 - $75,000
- ☐ $75,001 - $100,000
- ☐ $100,001 -$125,000
- ☐ $125,001 - $150,000
- ☐ $150,001 - $175,000
- ☐ $175,001 - $200,000
- ☐ More than $200,000
- ☐ I prefer not to answer

Q5a. Do you have health insurance that includes cover for your osteoporosis treatment?

- ☐ Yes
- ☐ No → GO TO Q5b

Q5b. What is the name of your plan/coverage option/insurance type? *(please ✓ all that apply)*

- ☐ Medicare
- ☐ Medicaid (or equivalent in your state)
- ☐ Medicare part D prescription drug plan
- ☐ Medicare medical savings account (MSA)
- ☐ Medicare advantage
- ☐ Employer provided/sponsored insurance
- ☐ Partner/family member employer insurance
- ☐ Privately arranged insurance
- ☐ Health insurance exchange plan
- ☐ Cobra (continuation coverage)
- ☐ Non-Medicare retired benefit
- ☐ Tricare/Veterans health care
- ☐ Other
- ☐ Don't know

SECTION F: ABOUT YOU (continued)

Q6a. Do you smoke? (Please ✓ one box only)

☐ Yes - currently → **Q6b. For how long for have you smoked?** _____months **OR** _____years

☐ Previously → **Q6c. How long ago did you stop smoking?** _____months **OR** _____years

☐ No

Q7. Over an average month, how often do you have a drink containing alcohol?
(Please ✓ one box only)

☐ Once a month or less

☐ 2-4 times per month

☐ 2-3 times per week

☐ 4 or more times per week

Q8. How many units of alcohol do you typically have on a day when you are drinking?
(1 unit is 1 small glass wine, ½ pint average strength beer or a single measure of spirit)
(Please ✓ one box only)

☐ 1 or 2

☐ 3 or 4

☐ 5 or 6

☐ 7 to 9

☐ 10 or more

Q9. As far as you know, have either of your parents ever had a hip fracture?
(Please ✓ one box only)

☐ Yes ☐ No ☐ Don't know

Q10. What is the main purpose of todays visit to your doctor?
(Please ✓ one box only)

☐ New symptom/ complaint ☐ To talk about my treatment ☐ To receive test results

☐ Regular or scheduled check up ☐ To participate in this study ☐ Other

END OF QUESTIONNAIRE

Thank you for participating in this survey. Your time is appreciated.

Signature Page for RAD-CLIN-000960 v1.0

Approval	Deepa Desai<br>Regulatory<br>22-May-2018 20:05:17 GMT+0000

Approval	Bruce Mitlak<br>Medical<br>22-May-2018 20:07:13 GMT+0000

Signature Page for RAD-CLIN-000960 v1.0

Adelphi Real World

INSIGHTS, EVIDENCE & LEADERSHIP SINCE 1995

8483 OSTEOPOROSIS CASE REPORT FORM

V7.0

May 2018

**Logic**	STANDARD PROGRAMMER INSTRUCTIONS – ALL RED TEXT<br>○ = radio button, therefore single choice. Mutually exclusive<br>○○○ = radio buttons shown in a rating scale, single select per row<br>☐ = multi choice button, do not allow to be selected in addition to a mutually exclusive option in same question (e.g. "○Not applicable")<br>If answers outside of range, show error message "Your response is outside the expected range. Please check your answer".<br>Ranges have been provided on relevant questions<br>Base provided for each question (e.g. <ASK ALL>)<br><br>PARTIAL DATES WILL BE COLLECTED IF FULL DATES ARE NOT AVAILABLE.

SCREENER

S1. How old is the patient?

Q type	Open numeric	VAR	
Range	18–100		
Base	All		
Logic			

S2. Is this patient female?

O	Yes
O	No <SCREEN OUT>

Q type	SC	VAR	
Range			
Base	All		
Logic	Screen out if 'Yes' not selected		

S3. Is this patient diagnosed with postmenopausal osteoporosis and considered to be at high risk of fractures by the treating physician?

O	Yes
O	No <SCREEN OUT>

Q type	SC	VAR	
Range			
Base	All		
Logic	Screen out if 'Yes' not selected		

S4. At enrollment, had this patient been prescribed SC TYMLOS® (abaloparatide) for at least one month?

O	Yes
O	No <SCREEN OUT>

Q type	SC	VAR	
Range			
Base	All		
Logic	Screen out if 'Yes' not selected<br>Link added for TYMLOS FDA label: https://www.accessdata.fda.gov/drugsatfda_docs/label/2017/208743lbl.pdf		

S5. Was this patient treated with Forteo® (teriparatide) as their previous treatment line prior to initiating treatment with TYMLOS (abaloparatide) for a period of at least one month?

O	Yes <ASSIGN TO COHORT II>
O	No <ASSIGN TO COHORT I>

Q type	SC	VAR	
Range			
Base	All		
Logic	Screen out if "don't know" selected		

	Link added for Forteo FDA label: https://www.accessdata.fda.gov/drugsatfda_docs/label/2009/021318s012lbl.pdf

S6. At the point of enrolment, was this patient participating in a clinical trial (including clinical trials that are not specific to osteoporosis)?

O	Yes <SCREEN OUT>
O	No

Q type	SC	VAR	
Range			
Base	All		
Logic	Screen out if 'No' not selected		

S7. Is this patient currently an outpatient?

O	Yes
O	No <SCREEN OUT>

Q type	SC	VAR	
Range			
Base	All		
Logic	Screen out if 'Yes' not selected		

S8. Has this patient been prescribed TYMLOS (abaloparatide) outside of the label indication?

O	Yes <SCREEN OUT>
O	No

Q type	SC	VAR	
Range			
Base	All		
Logic	Screen out if 'No' not selected		

S9. Is this patient diagnosed with any of the following conditions: Paget's disease, pre-existing hypercalcemia, or primary hyperparathyroidism??

O	Yes <SCREEN OUT>
O	No

Q type	SC	VAR	
Range			
Base	All		
Logic	Screen out if 'No' not selected		

S10. Does the patient have the necessary English skills to understand and complete their questionnaire?

O	Yes
O	No <SCREEN OUT>

Q type	SC	VAR	
Range			
Base	All		
Logic	Screen out if 'Yes' not selected		

S11. Has this patient voluntarily signed an informed consent form and returned their completed questionnaire?

O	Yes
O	No <SHOW ERROR MESSAGE>
O	Don't know <SHOW ERROR MESSAGE>

Q type	SC	VAR	
Range			
Base	All		
Logic	If 'Yes' not selected, show error message: 'Please wait for the patient to sign their informed consent form and complete their paper survey before proceeding'		

S12. Please indicate the date when the patient was enrolled in the study, i.e. the date when their patient questionnaire was completed?

	Calendar

Q type	Calendar	VAR	
Range	0–600 months (18000 days)		
Base	All		
Logic	Cannot be earlier than 01/07/2018 ~~31/05/2018~~<br>Cannot enter a date later than the date of completion		

S13. What is the specialty of this patient's main treating physician for their osteoporosis?

O	Primary care
O	Gynecologist / OB/GYN
O	Geriatrician
O	Rheumatologist
O	Endocrinologist
O	Orthopedist
O	Traumatologist
O	Other

Q type	SC	VAR	
Range			
Base	All		
Logic			

SECTION A: PATIENT DEMOGRAPHICS AND CLINICAL CHARACTERISTICS

Q1a. Patient's weight?

	Lbs

Q type	SC	VAR	Pat_weight
Range	70–630		
Base	All		
Logic			

Q1b. Patient's height?

	Feet		Inches

Q type	SC	VAR	Pat_weight
Range	2–7 feet, 0–11 inches		
Base	All		
Logic			

Q2a. Patient's race?

O	American Indian or Alaska Native
O	Asian
O	Black or African American
O	Native Hawaiian or other Pacific Islander
O	White
O	Other

Q type	SC	VAR	PatRace	
Range				
Base	All			
Logic				

Q2b. Patient's ethnicity?

O	Hispanic / Latino
O	Not Hispanic / Latino

Q type	SC	VAR	PatHispanic	
Range				
Base	All			
Logic				

Q3. When was this patient diagnosed with osteoporosis?

	Calendar
O	Don't know

Q type	Calendar or SC	VAR	
Range			

Base	All
Logic	Cannot enter a date later than the date of completion

Q4. In the 24 months prior to their osteoporosis diagnosis, was the patient exposed to chronic use of corticosteroids? *<Chronic use is defined as at least 7.5 mg of steroids for 90 days+>*

○	Yes
○	No
○	Don't know

Q type	SC	VAR	
Range			
Base	All		
Logic			

Q5. Please indicate the condition(s) for which this patient was treated with corticosteroid therapy prior to being diagnosed with osteoporosis

☐	Asthma
☐	COPD
☐	Allergic rhinitis
☐	Hives/eczema
☐	Arthritis
☐	Polymyalgia rheumatica (PMR)
☐	Neurological pain (e.g. Sciatica)
☐	Inflammatory bowel disease
☐	Lupus
☐	Multiple sclerosis
☐	Other

Q type	MC	VAR	
Range			
Base	All who selected 'Yes' at AQ4		
Logic			

Q6. Is the patient ***currently*** receiving chronic treatment with corticosteroids? *< Chronic use is defined as at least 7.5 mg of steroids for 90 days+>*

○	Yes
○	No
○	Don't know

Q type	SC	VAR	
Range			
Base	All		
Logic			

Q7. Are you aware of any of the following concomitant conditions for this patient?

	Current conditions<br>**Select all that apply**
**Cardiovascular conditions**	
Congestive heart failure (CHF)	☐
Peripheral vascular disease (PVD)	☐
Cerebrovascular disease	☐
Coronary heart disease (including myocardial ischemia-silent or acute)	☐
Cardiac dysrhythmia (including atrial fibrillation, atrial flutter and tachycardia [including SVT])	☐
Palpitations	☐
Obesity	☐
Hypertension	☐
**Endocrine disorder**	
Diabetes without chronic complications	☐
Diabetes with chronic complications	☐
Hyperthyroidism	☐
Hypothyroidism	☐
**GI conditions**	
Inflammatory bowel disease (IBD)	☐
Peptic Ulcer Disease	☐
Malabsorption syndrome	☐
Gastroesophageal reflux disease (GERD)	☐
**Cancer / hematological disorder**	
Hemiplegia	☐
Leukemia	☐
Lymphoma	☐
Tumor without metastasis	☐
Metastatic solid tumor	☐
Any tumor	☐
**Psychiatric disorder**	
Anxiety	☐
Depression	☐
Dementia	☐
**Renal disorder**	
Moderate/severe renal disease	☐
**Other**	
AIDS	☐
Connective tissue disease	☐
Mild liver disease	☐
Moderate or severe liver disease	☐
Chronic back pain (not related to osteoporosis)	☐
Osteoarthritis	☐
Periodontitis	☐
Rheumatoid arthritis	☐
Polymyalgia rheumatica	☐
Other rheumatological disease	☐
None	○

Q type	MC/SC	VAR	
Range			
Base	All		
Logic			

SECTION B: OSTEOPOROSIS THERAPIES

Q1. When was this patient first prescribed TYMLOS (abaloparatide)?

	Calendar
O	Don't know

Q type	Calendar	VAR	
Range	0–600 months (18000 days)		
Base			
Logic	Cannot be greater than time since diagnosis (AQ4). Must be at least 1 month before enrollment. Can't be pre-approval (soft prompt added for pre-April 2017)<br>Cannot enter a date later than the date of completion		

Q2. What is the scheduled duration of this patient's TYMLOS (abaloparatide) therapy?

	Months
O	Don't know

Q type	Calendar	VAR	
Range	0–60 months		
Base	All		
Logic	If respondent enter response greater than 48, show error message: 'This is outside the expected range. Are you sure?'<br>Cannot enter a date later than the date of completion		

Q3. To the best of your knowledge, does the patient take their TYMLOS (abaloparatide) medication as directed? [May need to be a question that can only be answered by the PI]

O	Yes
O	No
O	Too early to tell

Q type	SC	VAR	
Range			
Base	All		
Logic			

Q4. Was TYMLOS (abaloparatide) the patient's first pharmacological treatment for osteoporosis? <Cohort I patients only>

O	Yes
O	No
O	Don't know

Q type	SC	VAR	
Range			
Base	All who selected 'No' at S5		
Logic			

Q5. When was this patient first prescribed Forteo (teriparatide)? <Cohort II patients only>

	Calendar
O	Don't know

Q type	SC	VAR	
Range			
Base	All who selected 'Yes' at S5		
Logic	Must be earlier than date of TYMLOS initiation (BQ1)<br>Cannot enter a date later than the date of completion		

Q6. To the best of your knowledge, did the patient take Forteo (teriparatide) as directed? <Cohort II patients only> [May need to be a question that can only be answered by the PI]

O	Yes
O	No
O	Don't know

Q type	SC	VAR	
Range			
Base	All who selected 'Yes' at S5		
Logic			

Q7. What was the reason for this patient's switch from Forteo (teriparatide) to TYMLOS (abaloparatide)? <Cohort II patients only>

☐	Mandatory formulary switch to TYMLOS (abaloparatide)
☐	Patient requested a treatment change
☐	Lack of efficacy with Forteo (teriparatide)
☐	Patient had poor tolerability to Forteo (teriparatide)
☐	Financial / insurance reasons
☐	Other
O	Don't know

Q type	MC/SC	VAR	
Range			
Base	All who selected 'Yes' at S5		
Logic			

Q8. Was Forteo (teriparatide) the patient's first pharmacological treatment for osteoporosis? <Cohort II patients only>

O	Yes
O	No
O	Don't know

Q type	SC	VAR	
Range			
Base	All who selected 'Yes' at S5		
Logic			

IF TYMLOS (abaloparatide) (cohort 1) OR Forteo (teriparatide) (cohort II) was NOT the first pharmacological therapy, the following will be asked

For cohort I only:

Q9a. Prior to initiating TYMLOS (abaloparatide), what was this patient's previous treatment line? <Cohort I who have received a previous treatment>

Select one response

	Prescribed prior to TYMLOS (abaloparatide)
Alendronate Daily (e.g. Fosamax once daily)	○
Alendronate Weekly (e.g. Fosamax once weekly)	○
Alendronate + Vitamin D (e.g. Fosamax plus D)	○
Ibandronate Daily (e.g. Boniva)	○
Ibandronate Monthly (e.g. Boniva once monthly)	○
Ibandronate Quarterly IV (e.g. Boniva IV)	○
Calcitonin (subcutaneous injection)	○
Calcitonin (intranasal)	○
Hormone replacement therapy	○
Raloxifene daily (e.g. Evista)	○
Risedronate Daily (e.g. Actonel once daily)	○
Risedronate Weekly (e.g. Actonel once weekly)	○
Risedronate Monthly (e.g. Actonel once monthly)	○
Risedronate Weekly + Calcium (e.g. Actonel with calcium)	○
Risedronate Delayed Release (e.g. Atelvia)	○
Zoledronate Once yearly (e.g. Reclast)	○
Zoledronate Once 2-yearly (e.g. Reclast)	○
Denosumab (e.g. Prolia)	○
Other combination therapy	○
Other monotherapy	○

Q type	SC	VAR	
Range			
Base	All who selected 'No' at BQ4		
Logic			

Q9b. When did the patient start this previous treatment line?

	Calendar

Q type	SC	VAR	
Range	0–600 months (18000 days)		
Base	All who selected 'No' at BQ4		
Logic	Must be earlier than date of TYMLOS initiation (BQ1)<br>Cannot enter a date later than the date of completion		

Q9c. When did the patient stop this previous treatment line?

	Calendar

Q type	SC	VAR	
Range	0–600 months (18000 days)		
Base	All who selected 'No' at BQ4		
Logic	Cannot be earlier than date provided at BQ9b<br>Cannot be later than date provided at BQ1		

Q9d. Was this the patient's first pharmacological treatment for osteoporosis?

○	Yes
○	No
○	Don't know

Q type	SC	VAR	
Range			
Base	All who selected 'No' at BQ4		
Logic			

For Cohort II only

Q10a. Prior to initiating Forteo (teriparatide), what was this patient's previous treatment line? <Cohort II who have received a previous treatment>

Select one response

	Prescribed prior to TYMLOS (abaloparatide)
Alendronate Daily (e.g. Fosamax once daily)	○
Alendronate Weekly (e.g. Fosamax once weekly)	○
Alendronate + Vitamin D (e.g. Fosamax plus D)	○
Ibandronate Daily (e.g. Boniva)	○
Ibandronate Monthly (e.g. Boniva once monthly)	○
Ibandronate Quarterly IV (e.g. Boniva IV)	○
Calcitonin (subcutaneous injection)	○
Calcitonin (intranasal)	○
Hormone replacement therapy	○
Raloxifene daily (e.g. Evista)	○
Risedronate Daily (e.g. Actonel once daily)	○
Risedronate Weekly (e.g. Actonel once weekly)	○
Risedronate Monthly (e.g. Actonel once monthly)	○
Risedronate Weekly + Calcium (e.g. Actonel with calcium)	○
Risedronate Delayed Release (e.g. Atelvia)	○
Zoledronate Once yearly (e.g. Reclast)	○
Zoledronate Once 2-yearly (e.g. Reclast)	○
Denosumab (e.g. Prolia)	○
Other combination therapy	○
Other monotherapy	○

Q type	SC	VAR	

Range	
Base	All who selected ‘No’ at BQ8
Logic	

Q10b. When did the patient start this previous treatment line?

	Calendar

Q type	SC	VAR	
Range	0–600 months (18000 days)		
Base	All who selected ‘No’ at BQ8		
Logic	Must be earlier than date of Forteo initiation (BQ5)		

Q10c. When did the patient stop this previous treatment line?

	Calendar

Q type	SC	VAR	
Range	0–600 months (18000 days)		
Base	All who selected ‘No’ at BQ8		
Logic	Cannot be earlier than date provided at BQ10b<br>Cannot be later than date provided at BQ1		

Q10d. Was this the patient’s first pharmacological treatment for osteoporosis?

○	Yes
○	No
○	Don’t know

Q type	SC	VAR	
Range			
Base	All who selected ‘No’ at BQ8		
Logic			

Q11. When was this patient **first prescribed** a pharmacological treatment for osteoporosis? <only shown if applicable, *i.e. If previous questions indicate that TYMLOS (abaloparatide), Forteo (teriparatide) or the previous line were not the FIRST treatment.* >

	Calendar

Q type	SC	VAR	
Range	0–600 months (18000 days)		
Base	All who selected ‘No’ at BQ4 (cohort I) OR BQ8 (cohort II)		
Logic	Must be earlier than date provided at BQ8bi (cohort I) or BQ9bi (cohort II)<br>Cannot enter a date later than the date of completion		

Q12. Which of the following therapies has this patient ever received for their osteoporosis which have not previously been specified? *<Only shown if applicable. I.e. If previous questions indicate that TYMLOS (abaloparatide), Forteo (teriparatide) or the previous line were not the FIRST treatment. >*

Select all that apply

	Ever received
Alendronate Daily (e.g. Fosamax once daily)	☐
Alendronate Weekly (e.g. Fosamax once weekly)	☐
Alendronate + Vitamin D (e.g. Fosamax plus D)	☐
Ibandronate Daily (e.g. Boniva)	☐
Ibandronate Monthly (e.g. Boniva once monthly)	☐
Ibandronate Quarterly IV (e.g. Boniva IV)	☐
Calcitonin (subcutaneous injection)	☐
Calcitonin (intranasal)	☐
Hormone replacement therapy	☐
Raloxifene daily (e.g. Evista)	☐
Risedronate Daily (e.g. Actonel once daily)	☐
Risedronate Weekly (e.g. Actonel once weekly)	☐
Risedronate Monthly (e.g. Actonel once monthly)	☐
Risedronate Weekly + Calcium (e.g. Actonel with calcium)	☐
Risedronate Delayed Release (e.g. Atelvia)	☐
Zoledronate Once yearly (e.g. Reclast)	☐
Zoledronate Once 2-yearly (e.g. Reclast)	☐
Denosumab (e.g. Prolia)	☐
Other combination therapy	☐
Other monotherapy	☐

Q type	MC	VAR	
Range			
Base	All who selected 'No' at BQ4 (cohort I) OR BQ8 (cohort II)		
Logic			

END OF SECTION

SECTION C: DIAGNOSIS AND MONITORING OF OSTEOPOROSIS

Q1. Which of the following best describes the circumstances that lead to this patient's diagnosis of osteoporosis?

☐	Routine screening (i.e. pre-emptive screening due to age and/or risk status)
☐	Patient presented with risk factors (e.g. family history, comorbid condition that increase risk status/ prior medications that increased risk status)
☐	Patient presented with a fragility fracture
☐	Patient presented with a traumatic fracture
☐	Patient presented with symptoms associated with osteoporosis, excluding fractures (e.g. reduced height, stooped posture)
☐	Don't know
☐	Other

Q type	MC	VAR	
Range			
Base	All		

Q2. Which of the following tests were used to diagnose this patient with osteoporosis?

☐	Central DXA scan
☐	Peripheral DXA scan
☐	FRAX risk assessment
☐	Conventional X-ray
☐	Magnetic resonance imaging (MRI) scan
☐	Computed tomography (CT) scan
☐	Ultrasound
☐	Vertebral fracture analysis (VFA)
☐	Other

Q type	MC	VAR	
Range			
Base	All		
Logic			

Q3. Were any of the initial tests that aided or led to a diagnosis of osteoporosis originally conducted to evaluate a different condition? I.e. Was osteoporosis entirely or partially discovered due to investigations for a separate condition?

○	Yes
○	No
○	Don't know

Q type	SC	VAR	
Range			
Base	All		
Logic			

Q4. Which of the following tests have been used to assess / monitor this patient since diagnosis?

☐	Central DXA scan
☐	Peripheral DXA scan
☐	FRAX risk assessment
☐	Conventional X-ray
☐	Magnetic resonance imaging (MRI) scan
☐	Computed tomography (CT) scan
☐	Ultrasound
☐	Vertebral fracture analysis (VFA)
☐	Other

Q type	MC	VAR	
Range			
Base	All		
Logic			

Q5a. Which of the following sites were tested during the central DXA scan to aid diagnosis?

☐	Femoral neck
☐	Total hip
☐	Lumbar spine
☐	Radius
☐	Other area
○	Don’t know

Q type	MC/SC	VAR	
Range			
Base	All who selected ‘Central DXA scan’ at CQ2		
Logic			

Q5b. Please specify the available T scores from the central DXA scan conducted at diagnosis

**Site**	**T-score**	**Don’t know**
Femoral neck		○
Total hip		○
Lumbar spine		○
Radius		○
Other area		○
Don’t know		○

Q type	Open numeric/SC	VAR	
Range	~~<~~-10 to 0		
Base	All		
Logic	Only show rows selected at DQ5a		

Q5c. Please specify the date when central DXA scan at diagnosis was conducted

	Calendar
○	Don't know

Q type	Calendar or SC	VAR	
Range	0–600 months (18000 days)		
Base	All who selected 'Central DXA scan' at CQ2		
Logic	Cannot be earlier than date of diagnosis (AQ3)<br>Cannot enter a date later than the date of completion		

Q6. How many times has this patient received a central DXA scan in the last 24 month? (**Excluding** the scan done for diagnosis if this was within the last 24 months).

Q type	Open numeric	VAR	
Range	0–35		
Base	All who selected code 1 CQ4		
Logic			

Q7a. Which of the following sites were tested at the most recent central DXA scan?

☐	Femoral neck
☐	Total hip
☐	Lumbar spine
☐	Radius
☐	Other area
○	Don't know

Q type	Open numeric	VAR	
Range			
Base	All who selected code 1 CQ4		
Logic			

Q7b. Please specify the available T scores from the most recent central DXA scan conducted

**Site**	**T-score**	**Don't know**
Femoral neck		○
Total hip		○
Lumbar spine		○
Radius		○
Other area		○
Don't know		○

Q type	Open numeric/SC	VAR	
Range	~~<~~-10 to 0		
Base	All who selected code 1 CQ4		
Logic	Only show rows selected at DQ7a		

Q7c. Please specify the date when the most recent central DXA scan was conducted

	Calendar
○	Don't know

Q type	Calendar or SC	VAR	
Range	0–600 months (18000 days)		
Base	All who selected code 1 CQ4		
Logic	Cannot enter a date later than the date of completion		

Q8. Please specify this patient's FRAX assessment score conducted at diagnosis

**The ten-year probability of fracture…**	**%**	**Don't know**
Major osteoporotic fracture with BMD	_____	○
Major osteoporotic fracture without BMD	_____	○
Hip fracture with BMD	_____	○
Hip fracture without BMD	_____	○
Unknown		○

Q type	Open numeric/SC	VAR	
Range	0–100%		
Base	All who selected code 3 at CQ2		
Logic			

Q9. Have bone turn over markers (BTMs) been assessed for this patient at any of the following points?

Select all that apply

☐	At diagnosis
☐	When the patient's current treatment with TYMLOS (abaloparatide) was first initiated
☐	To monitor the patient's current osteoporosis treatment with TYMLOS (abaloparatide) (may be too soon to answer)
○	None of the above

Q type	MC/SC	VAR	
Range			
Base	All		
Logic			

Q10. Thinking about this patient's last treatment prior to initiating TYMLOS (abaloparatide), were BTMs used to assess this previous line of treatment? *<will only show when applicable, i.e. those with treatment history prior to TYMLOS (abaloparatide)>*

☐	When the previous treatment was initiated
☐	To monitor the patient's previous treatment
○	None of the above

Q type	SC	VAR	
Range			
Base	All who selected 'No' at BQ4 (cohort I) OR BQ8 (cohort II)		
Logic			

<If monitoring selected>

Q11. When were BTMs assessed for this patient in relation to their previous treatment?

Select all that apply

☐	Within 3 months of treatment initiation
☐	Within 4 – 6 months of treatment initiation
☐	7+ months after treatment initiation

Q type	MC	VAR	
Range			
Base	All who selected code 2 at CQ10		
Logic			

Q12. Which BTMs have ever been assessed in this patient?

	**Formation**		**Resorption**
☐	Serum total alkaline phosphatase	☐	Urinary hydroxyproline
☐	Serum bone–specific alkaline phosphatase	☐	Urinary total pyridinoline (PYD)
☐	Serum osteocalcin	☐	Urinary free deoxypyridinoline (DPD)
☐	Serum type 1 procollagen (C-terminal/N-terminal): C1NP or P1NP	☐	Urinary collagen type 1 cross-linked N-telopeptide (NTX)
		☐	Urinary or serum collagen type 1 cross-linked C-telopeptide (CTX)
		☐	Bone sialoprotein (BSP)
		☐	Tartrate-resistant acid phosphatase 5b

Q type	MC	VAR	
Range			
Base	All who selected codes 1–3 at CQ9 or codes 1–2 at CQ10		
Logic	Only show number of rows entered at DQ4b		

Q13. Are BTM tests currently reimbursed for this patient?

○	Yes
○	No
○	Don't know

Q type	SC	VAR	
Range			
Base	All who selected codes 1–3 at CQ9 or codes 1–2 at CQ10		
Logic			

Q14a. Have the results of any BTM assessments been communicated to the patient? [Question can only be answered by the PI]

○	Yes
○	No
○	Don't know

Q type	SC	VAR	
Range			

Base	All who selected codes 1–3 at CQ9 or codes 1–2 at CQ10
Logic	

Q14b. To the best of your knowledge, have any of the BTM results influenced the patient to persist with the therapy? [Question can only be answered by the PI]

○	Yes
○	No
○	Don't know

Q type	SC	VAR	
Range			
Base	All who selected codes 1–3 at CQ9 or codes 1–2 at CQ10		
Logic			

Q15. In your professional opinion, what is your **subjective** view of this patient's current severity level for osteoporosis? [Question can only be answered by the PI]

○	Mild
○	Moderate
○	Severe
○	Very severe

Q type	SC	VAR	
Range			
Base	All		
Logic			

END OF SECTION

SECTION D: FRACTURE HISTORY AND RELATED HOSPITALIZATIONS

Q1a. How many of the following osteoporotic fracture events did this patient suffer **one year prior** to initiation of TYMLOS (abaloparatide) therapy?

Enter a number. If no fractures were suffered, please enter '0'

Vertebral fractures (i.e. thoracolumbar region)	
Non-vertebral fractures (i.e. forearm, humerus, sacrum, pelvis, hip, femur or tibia)	

Q type	Open numeric	VAR	
Range	0–20 per row		
Base	All		
Logic			

Q1b. Of these osteoporotic fractures suffered prior to initiation of TYMLOS (abaloparatide) therapy, how many have required hospitalization?

Enter a number. If no fractures required hospitalization, please enter '0'

Vertebral fractures (i.e. thoracolumbar region)	
Non-vertebral fractures (i.e. forearm, humerus, sacrum, pelvis, hip, femur or tibia)	

Q type	Open numeric	VAR	
Range	0–20 per row		
Base	Only show rows with a response > 0 at DQ1a		
Logic	Each row cannot be greater than @ DQ1a		

Q2a. How many of the following osteoporotic fracture events has this patient suffered **since initiating TYMLOS (abaloparatide) therapy**?

Enter a number. If no fractures were suffered, please enter '0'

Vertebral fractures (i.e. thoracolumbar region)	
Non-vertebral fractures (i.e. forearm, humerus, sacrum, pelvis, hip, femur or tibia)	

Q type	Open numeric	VAR	
Range	0–20 per row		
Base	Only show rows with a response > 0 at DQ1a		
Logic	Each row cannot be greater than @ DQ1a		

Q2b. Please specify the date(s) of this patient's vertebral fracture(s) since initiating TYMLOS (abaloparatide) therapy

Please enter the date the fracture was diagnosed. If multiple fractures have occurred, please enter all known dates starting with the most recent

**Most recent**	<Calendar>	○ Don't know
**2nd most recent**	<Calendar>	○ Don't know
**3rd most recent**	<Calendar>	○ Don't know

Q type	Calendar/SC per row	VAR	
Range	0–600 months (18000 days)		
Base	All		
Logic	Only show number of rows entered at DQ2a<br>Cannot enter a date later than the date of completion		

Q2c. Please specify the date/s of this patient's non-vertebral fractures since initiating TYMLOS (abaloparatide) therapy

Please enter the date the fracture was diagnosed. If multiple fractures have occurred, please enter all known dates starting with the most recent

**Most recent**	<Calendar>	O Don't know
**2nd most recent**	<Calendar>	O Don't know
**3rd most recent**	<Calendar>	O Don't know

Q type	Open numeric/SC per row	VAR	
Range			
Base	All		
Logic	Only show number of rows entered at DQ2a<br>Cannot enter a date later than the date of completion		

Q2d. Of these osteoporotic fractures **since initiating TYMLOS (abaloparatide) therapy,** how many have required **hospitalization**?

Enter a number. If no fractures required hospitalization, please enter '0'

Vertebral fractures (i.e. thoracolumbar region)	
Non-vertebral fractures (i.e. forearm, humerus, sacrum, pelvis, hip, femur or tibia)	

Q type	Open numeric	VAR	
Range	0–20 per row		
Base	Only show rows with a response > 0 at DQ2a		
Logic	Each row cannot be greater than @ DQ2a		

END OF CRF

SITE LEVEL QUESTIONS

(Completed once, not per patient)

Q1. Is your site classed as an academic/ teaching hospital?

○	Academic/ teaching hospital
○	Other

Q2. Is your site independent or part of a network?

○	Independent
○	System/ network

Q3a. Which of the following educational programs are you able to offer your osteoporosis patients?

☐	Nutritional support
☐	Exercise programs
☐	Disease education
☐	Drug/ treatment education
☐	Fall prevention programs
☐	Other
○	None

Q3b. Does your site provide any of the following support groups for your osteoporosis patients?

☐	Live support groups/ advocacy
☐	Social media/ online support groups
☐	Financial assistance/ insurance coverage support for treatment
○	None

Q4. When your patients are prescribed a central DXA scan, where does this normally take place?

○	On site
○	Referral to another center

Q5. At your center, is there a specific protocol for osteoporosis or treatment that you have to follow?

○	Yes
○	No
